# Complete Genome Sequence of *Campylobacter jejuni* subsp. *jejuni* ATCC 35925

**DOI:** 10.1128/genomeA.00743-17

**Published:** 2017-07-27

**Authors:** Susan P. Gardner, Kevin J. Kendall, Michael E. Taveirne, Jonathan W. Olson

**Affiliations:** a Department of Biological Sciences, North Carolina State University, Raleigh, North Carolina, USA; b MacVector, Inc., Cary, North Carolina, USA; c Department of Microbiology and Immunology, University of Michigan Medical School, Ann Arbor, Michigan, USA

## Abstract

Here, we report the complete genome sequence of *Campylobacter jejuni* ATCC 35925, an avian isolate from Sweden. The genome gives insight into the ATCC 35925 strain’s remarkable ability to tolerate copper and its permissiveness to plasmid transformation.

## GENOME ANNOUNCEMENT

*Campylobacter jejuni* is an important foodborne pathogen, often associated with the consumption or mishandling of raw or undercooked poultry. Campylobacteriosis causes gastroenteritis in humans and is an antecedent to Guillain-Barré syndrome and postinfectious irritable bowel syndrome (PI-IBS) (1–3).

In recent years, there has been much interest in replacing antibiotics in animal feed with copper, which acts as both an antimicrobial agent and a growth promoter (4). *C. jejuni* ATCC 35925 is an avian strain isolated in Sweden (5) that is unusual in that it has greater resistance to copper than most type strains of *C. jejuni* (our unpublished data). Work has been done in *C. jejuni* to elucidate the importance of copper resistance mechanisms (6) but in a strain (NCTC 11168) that is much more sensitive to copper. Additionally, *C. jejuni* ATCC 35925 is an appealing laboratory strain because it is very permissive to plasmid transformation.

*C. jejuni* ATCC 35925 genomic DNA was extracted using the Epicentre MasterPure Complete DNA and RNA purification kit (Illumina), and DNA concentrations were determined using the Qubit double-stranded DNA (dsDNA) BR assay kit (Invitrogen). Whole-genome sequencing was performed using Illumina paired-end 300-cycle reads on the MiSeq system technology platform. A total of 3,297,084 raw paired-end reads were assembled using the MacVector 15.1 (MacVector, Inc., Cary, NC) implementation of the *de novo* assembler Velvet (7), with a k-mer value of 269 resulting in 26 contigs with an average read depth of >160 bp and an *N*_*50*_ of >159,227 bp. The contigs were ordered against the closely related genome of *C. jejuni* subsp. *jejuni* IA3902 (GenBank accession no. CP001876), and the repeat sequences at the ends of the contigs were resolved using MacVector’s Align to Reference and Align To Folder tools. The final 1,618,194-bp circular genome was annotated using the NCBI Prokaryotic Genome Annotation Pipeline (http://www.ncbi.nlm.nih.gov/genome/annotation_prok).

Additional analysis using MacVector revealed significant differences between this genome and a previously published genome sequence of strain 35925 (originally 35925B2, GenBank accession no. CP010906). There are 28 single nucleotide polymorphisms and 4 insertions/deletions varying in size between 10 bp and 272 bp. In addition, the two genomes have exchanged a 496-bp internal region between two versions of *pseD* (B5D75_RS06330 and B5D75_RS06380). It is not clear if the observed differences are due to sequencing/assembly issues or variations accumulated during passage within different laboratories.

This genome gives insight into some of the phenotypic characteristics that make 35925 attractive for our research. The high transformation efficacy is due to the fact that this strain lacks the genes that encode the three restriction endonucleases found in NCTC 11168, Cj1551c, Cj0031, and Cj1461 (8). We hypothesize that the higher copper resistance of 35925 versus 11168 is due to the presence of a gamma-glutamyl transpeptidase (B5D75_RS00190) and a thioredoxin (B5D75_RS00180) not found in NCTC 11168. These proteins work together to modulate the internal redox equilibrium, which would dampen the pro-oxidative stress properties of copper.

### Accession number(s).

This genome project has been deposited in GenBank under the accession number CP020045. The BioProject designation for this project is PRJNA378565.
